# The Transformation of Empathic Care in the Digital Age: A Scoping Review on Digital Empathy from a Nursing Perspective

**DOI:** 10.3390/healthcare14142042

**Published:** 2026-07-08

**Authors:** Aytolan Yıldırım, Gizem Açıkgöz, Leman Kutlu

**Affiliations:** Department of Nursing, Faculty of Health Sciences, Atlas University, Istanbul 34408, Türkiye; aytolan.yildirim@atlas.edu.tr (A.Y.); leman.kutlu@atlas.edu.tr (L.K.)

**Keywords:** digital empathy, nursing, digital health, telehealth, artificial intelligence, person-centred care, technology-mediated care, scoping review

## Abstract

Background/Objectives: The digitalization of healthcare has transformed nursing care and stimulated growing interest in digital empathy. However, the literature remains conceptually diverse and fragmented. This scoping review aimed to map the nursing literature on digital empathy, examining its definitions, its role in nurse–individual interactions, the factors that support or hinder it, and the research gaps in this field. Methods: A scoping review was conducted in accordance with the Joanna Briggs Institute methodology and reported following the PRISMA-ScR guideline. Electronic searches were performed in PubMed, CINAHL, Scopus, and Web of Science for studies published between 2000 and 2026. Eligible studies addressed digital empathy in nursing. Data were extracted and synthesized using descriptive and thematic analysis. Results: Thirty-three studies published between 2009 and 2026 met the inclusion criteria. Thematic synthesis identified four overarching themes: (1) definition and conceptualization of digital empathy, (2) its role in nurse–individual interaction and nursing care experience, (3) factors supporting and hindering digital empathy in technology-mediated nursing care, and (4) research gaps and future needs. The findings indicate that digital empathy is increasingly recognized as an important component of technology-mediated nursing care, although conceptual definitions remain heterogeneous and theoretical development limited. Conclusions: Digital empathy is an emerging and evolving concept that may enhance compassionate, person-centred, and relational nursing care in digital healthcare environments. These findings may inform future conceptual, educational, clinical, and research developments related to digital empathy in nursing. Registration: This scoping review was prospectively registered with the Open Science Framework on 9 April 2026 (Registration DOI: 10.17605/OSF.IO/B2JN8).

## 1. Introduction

Digitalization has transformed healthcare delivery and communication worldwide. This transformation is evident in clinical communication, patient monitoring, health education, and decision-making processes [1,2,3,4]. In nursing, it is reflected in the growing use of telehealth, remote monitoring, virtual care models, and AI-supported services [1,3]. The COVID-19 pandemic further accelerated the adoption of these technologies and underscored their importance in maintaining continuity of care [5]. Despite these advances, concerns remain about preserving therapeutic relationships, meaningful communication, and empathic care in technology-mediated environments [2,5,6].

Empathy is central to nursing practice, underpinning person-centred care, therapeutic relationships, effective communication, trust, and positive patient outcomes [7,8,9]. It is commonly defined as the ability to understand patients’ perspectives and emotions, convey this understanding, and respond with compassion [8]. Through empathic care, nurses can better address patients’ holistic needs and provide individualized support [6,10]. Traditionally, empathy has been expressed through verbal and non-verbal communication, including active listening, tone of voice, and facial expressions [1,9]. However, increasing digitalization has reshaped how empathic relationships are established and maintained, prompting growing interest in how empathy can be conveyed and experienced in technology-mediated care environments [1,4,11].

In response to the digitalization of healthcare, digital empathy has emerged as an evolving concept. It broadly refers to the ability to understand, communicate, and respond to others’ emotions and needs through technology-mediated interactions while maintaining meaningful human connection [1,12]. Rather than replacing traditional empathy, digital empathy is increasingly understood as an extension of empathic nursing practice. It seeks to preserve the relational foundations of empathy, including emotional understanding, compassionate communication, therapeutic presence, and person-centred care within digitally mediated environments. Related concepts such as e-empathy, digital compassion, virtual empathy, and techno-caritas reflect its evolving nature. Nevertheless, these terms are often used interchangeably despite differences in theoretical emphasis and scope, contributing to ongoing conceptual ambiguity. Importantly, digital empathy is recognized as vital for fostering engagement, collaboration, and supportive interactions in digital environments [13].

Within nursing, digital empathy has been explored across diverse contexts, including education, telehealth, mental health, oncology, community, and older adult care. It has also been examined in relation to telenursing, digital communication platforms, and AI-supported healthcare systems [4,14,15,16,17]. Although digital technologies enhance communication, accessibility, continuity of care, and patient engagement, challenges related to emotional connection and therapeutic presence remain [2,4]. Consequently, the conceptual boundaries between traditional and digital empathy remain insufficiently defined, and no universally accepted framework currently exists to distinguish their shared and context-specific attributes. As a result, the conceptualization of digital empathy in nursing remains fragmented and lacks a consistent theoretical foundation [12].

Concerns remain regarding the preservation of empathy, person-centred care, and human connection within increasingly digital healthcare environments. These concerns are particularly evident in relation to depersonalisation, digital exclusion, technological barriers, and the algorithmic mediation of care [18,19,20]. At the same time, digital empathy is increasingly viewed as a capability that supports compassionate and relational nursing care in technology-mediated settings [14,15]. Despite growing interest, the literature remains dispersed across disciplines, care settings, and technologies, limiting a comprehensive understanding of how digital empathy has been conceptualized and addressed in nursing [1,2,4,11,12,13,14]. Therefore, this scoping review aimed to map the nursing literature on digital empathy and to clarify its definitions, role in nurse–individual interactions, influencing factors, and existing research gaps.

## 2. Materials and Methods

### 2.1. Study Design

This study was designed as a scoping review. It was conducted in accordance with the Joanna Briggs Institute (JBI) methodology for scoping reviews and reported following the PRISMA-ScR guideline [21,22,23]. The protocol was prospectively registered with the Open Science Framework on 9 April 2026 (Registration DOI: 10.17605/OSF.IO/B2JN8).

Scoping review methodology was originally proposed by Arksey and O’Malley [24] and later refined by Levac et al. [25]. For this review, the JBI methodology was chosen because it offers a comprehensive and structured framework for study selection, data extraction, evidence mapping, and synthesis. At the same time, it preserves the exploratory nature of scoping reviews [21,22,24,25].

### 2.2. Aim and Review Questions

The aim of this scoping review was to map the nursing literature on digital empathy. Specifically, it sought to clarify how the concept has been defined, how it has been addressed in nurse–individual interactions, the factors that support or hinder digital empathy, and the research gaps in this field.

This scoping review addressed the following questions:

1. How is the concept of digital empathy defined and conceptualized in the nursing literature?
2. How is digital empathy addressed in the context of nurse–individual interaction and nursing care experience?
3. What factors support or hinder digital empathy in technology-mediated nursing care processes?
4. What are the prominent research gaps and future research needs in the literature on digital empathy?

### 2.3. Eligibility Criteria

The eligibility criteria were structured according to the Population–Concept–Context (PCC) framework recommended by the Joanna Briggs Institute (JBI) for scoping reviews [21,23].

- Population: Nurses, nursing students, and individuals receiving nursing care were included, without restrictions on age, sex, clinical field, professional experience, or practice duration.
- Concept: The focus was digital empathy, encompassing empathic communication in digital environments, technology-mediated empathic nursing care, and related constructs such as therapeutic communication, caring presence, trust, emotional support, and human-centred care in digital settings.
- Context: The context included digital or technology-mediated nursing care processes, such as telehealth, remote care, eHealth, online nursing interactions, digital communication platforms, telephone-based nursing, video consultations, mobile health applications, AI-supported care, chatbots, and other digital tools used in nursing practice.

By contrast, studies without a nursing context, those focusing solely on technical system development, and those that did not address empathy, empathic communication, or human-centred care were excluded.

### 2.4. Types of Evidence Sources

This scoping review included quantitative, qualitative, and mixed-methods primary studies aligned with the review questions and the PCC framework. Eligible designs comprised experimental, quasi-experimental, descriptive, cross-sectional, observational, and pilot studies. Qualitative studies such as phenomenological, descriptive qualitative, interview-based, and focus group research were also considered. In addition, conceptual, theoretical, methodological, and review articles were included when relevant to the scope of the review. Broader inclusion was appropriate given that digital empathy is an emerging and evolving concept in nursing literature. Conversely, letters to the editor, conference abstracts, book chapters, theses, and grey literature were excluded.

### 2.5. Search Strategy

The search strategy was developed according to the PCC framework and included terms related to nursing, empathy, and technology-mediated care. Controlled vocabulary and free-text keywords were used, and the terms were adapted to the indexing structure of each database. Boolean operators “AND” and “OR” were applied to combine the search blocks.

Electronic searches were conducted in PubMed, CINAHL, Scopus, and Web of Science. The strategy included combinations such as “nurs*,” “nursing student*,” “nursing care,” “nurse-patient relation*,” “empath*,” “empathic communication,” “empathic care,” “telehealth,” “telemedicine,” “digital,” “online,” “virtual,” “remote care,” “digital communication,” “online communication,” and “computer-mediated communication.” In Web of Science, additional terms such as “digital empathy,” “e-empathy,” “eHealth,” “mHealth,” “mobile health,” “remote monitoring,” and “videoconferenc*” were used to increase sensitivity. Full search strategies for all databases are provided in Table 1.

The selected databases were chosen to ensure broad coverage of nursing and interdisciplinary healthcare literature relevant to the review questions. As the objective was to identify studies explicitly addressing digital empathy and related concepts, the search strategy was intentionally centred on empathy-related terminology combined with digital health and communication terms. Although additional databases and broader caring-related concepts might offer complementary perspectives, the chosen sources and terms were considered most appropriate for identifying evidence relevant to the scope and objectives of this review. Finally, studies indexed by the date of the final search, including online ahead-of-print articles, were considered eligible if they met the predefined inclusion criteria.

The reference lists of the included studies were screened to identify additional relevant sources. Full-text publications in Turkish or English, published from 2000 onward, were considered eligible. The year limit was applied because digital health practices and technology-mediated nursing care have become increasingly visible in the nursing literature since 2000. The language restriction was applied to ensure that the review team could reliably screen, extract, and interpret data. Limiting the review to these languages ensured accuracy and consistency while minimizing translation errors. Finally, the searches were conducted between 10 April and 25 April 2026, with the final search completed on 25 April 2026.

### 2.6. Source of Evidence Selection

All records identified through the database searches were screened after duplicates were removed. Study selection followed two sequential stages in accordance with the Joanna Briggs Institute (JBI) methodology and PRISMA-ScR recommendations [21,22,23].

Records retrieved from PubMed, Scopus, CINAHL, and Web of Science were exported and merged into a single Microsoft Excel spreadsheet. Duplicates were identified manually by comparing titles, authors, journal names, and publication years, and removed before screening commenced. In the first stage, titles and abstracts were screened against the eligibility criteria to identify potentially relevant studies. In the second stage, the full texts of the remaining studies were assessed for eligibility. Screening and selection were conducted independently by two reviewers, while a third reviewer oversaw the process, reviewed decisions when necessary, and provided methodological support. Disagreements were resolved through discussion, with consensus reached among the review team. The study selection process is illustrated in the PRISMA-ScR flow diagram.

### 2.7. Data Extraction

Data were extracted using a standardized form developed in line with the study’s aim and review questions. The process was conducted independently by two reviewers, with a third reviewer overseeing the procedure, reviewing decisions when necessary, and providing methodological support.

The extraction form captured information on authors, year of publication, country, study design, population and participant characteristics, nursing context, digital care setting or technology, definitions and conceptualizations of digital empathy, its role in nurse–individual interactions and nursing care experiences, factors supporting or hindering digital empathy, research gaps, and key findings. The form was refined iteratively to ensure comprehensive data capture.

### 2.8. Data Analysis and Presentation

The extracted data were analyzed using both descriptive and thematic approaches. Descriptive analysis summarized the characteristics of the included studies, such as publication year, country, study design, population, and digital care context. Findings were then synthesized narratively to address the review questions and to map the current state of knowledge on digital empathy in nursing. Review, conceptual, and theoretical articles were used to support conceptual mapping rather than to provide primary empirical evidence. During synthesis, findings from these sources were compared with those of primary studies, and recurring concepts were integrated to minimize duplication from overlapping publications.

Thematic analysis was employed to identify recurring patterns, concepts, and relationships across the included studies. Two reviewers independently coded the extracted data using an inductive approach. Initial codes were compared, refined through regular discussions, and grouped into broader categories before being synthesized into overarching themes. Discrepancies in coding or theme development were resolved through discussion and consensus, with methodological oversight provided by a third reviewer when required. This iterative process strengthened the credibility of the thematic synthesis. Themes were developed inductively from the extracted data and organized according to the review questions. The synthesis was structured around four themes: (1) definition and conceptualization of digital empathy, (2) its role in nurse–individual interaction and nursing care experience, (3) factors influencing digital empathy in technology-mediated nursing care, and (4) research gaps and future needs. The findings related to these themes are presented in the Section 3 through narrative synthesis and supporting tables.

### 2.9. Quality Appraisal

In line with the purpose and methodology of a scoping review, a formal methodological quality appraisal of the included studies was not conducted [21,24,25]. Instead, the aim of this review was to map the scope, characteristics, and conceptual dimensions of the existing literature on digital empathy from a nursing perspective, rather than to evaluate the methodological quality of individual studies or determine the effectiveness of specific interventions.

## 3. Results

### 3.1. Study Selection

**Figure 1:**
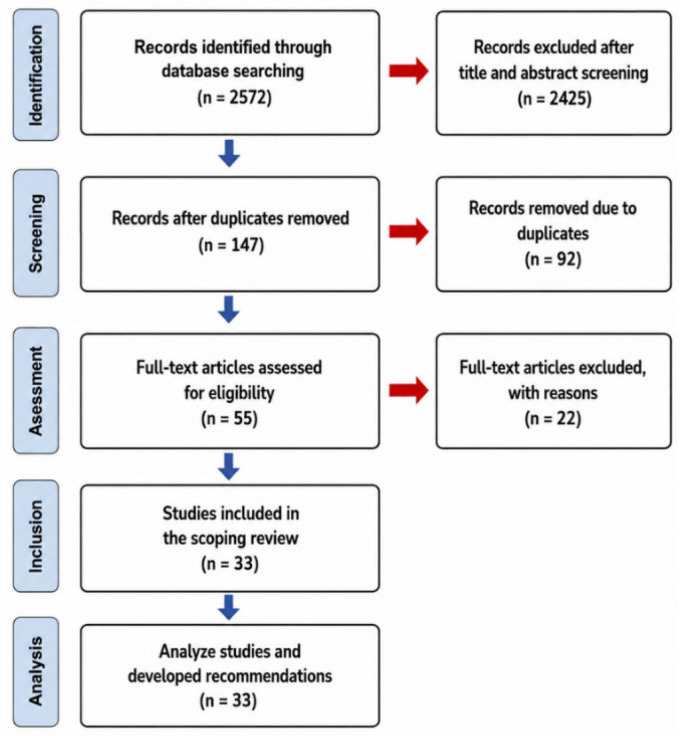
PRISMA-ScR flow diagram of the study selection process.

A total of 2572 records were identified through database searching: PubMed (*n* = 465), Scopus (*n* = 406), CINAHL (*n* = 562), and Web of Science (*n* = 1139). After title and abstract screening, 2425 records were excluded, leaving 147 potentially eligible studies. Among these, 92 duplicates were identified and removed, resulting in 55 studies for full-text assessment. Following full-text review, 22 studies were excluded for the following reasons: (a) no nursing context or personnel (*n* = 8); (b) primary focus on technical system development without examining relational or empathic aspects of care (*n* = 6); (c) empathy absent or only tangentially mentioned (*n* = 4); (d) conference abstract or editorial lacking substantive content (*n* = 2); and (e) full text unavailable despite contacting authors (*n* = 2). Ultimately, 33 studies were included in the final synthesis. The study selection process is illustrated in Figure 1.

### 3.2. Characteristics of Included Studies

The 33 studies were published between 2009 and 2026, with a noticeable increase in the last five years, reflecting growing interest in digital empathy and technology-mediated nursing care. The studies originated from diverse regions, including North America, Europe, the Middle East, and Asia. A wide range of methodological approaches were represented, including qualitative, quantitative, and mixed-methods studies, as well as concept analyses, literature reviews, scoping reviews, discussion papers, and theoretical publications. The included studies covered nursing contexts such as telehealth, telenursing, oncology, palliative care, primary care, community and home care, geriatric nursing, psychiatric nursing, intensive care, chronic disease management, nursing education, and AI-supported nursing practice. Participants included nurses, nursing students, patients, family caregivers, healthcare professionals, and other stakeholders involved in digital health services. The characteristics of the included studies are presented in Table 2.

A broad range of digital technologies were examined, including telephone-based services, videoconferencing, telehealth consultations, telemonitoring systems, mobile health applications, online communication platforms, conversational agents, chatbots, AI-supported systems, and other digital health tools. Across the included studies, empathy was described through diverse but related concepts such as digital empathy, e-empathy, therapeutic communication, compassionate care, trust, authenticity, relational presence, emotional support, and person-centred care. This diversity highlights the evolving, multidimensional nature of digital empathy in nursing. The thematic synthesis of the included studies resulted in four overarching themes: (1) definition and conceptualization of digital empathy, (2) digital empathy in nurse–individual interactions and nursing care experiences, (3) factors supporting and hindering digital empathy in technology-mediated nursing care, and (4) research gaps and future needs.

The included studies represented diverse methodological designs, reflecting the emerging and interdisciplinary nature of digital empathy research in nursing. Qualitative studies constituted the largest proportion of the evidence base, followed by theoretical, conceptual, and discussion papers, while quantitative studies, reviews, and concept analyses were less frequently represented. An overview of methodological characteristics is presented in Table 3.

In terms of care contexts, telehealth and telenursing were the most frequently investigated, followed by nursing education, AI-supported nursing care, oncology, and community or home-based care. Smaller numbers of studies addressed mental health nursing, primary care, intensive care, chronic disease management, and culturally tailored digital care interventions. Overall, the evidence indicates that digital empathy has been explored predominantly in technology-mediated communication and remote care settings, whereas several clinical contexts remain comparatively underrepresented.

### 3.3. Synthesized Conceptual Definition of Digital Empathy

The synthesis of the included studies demonstrated that digital empathy is consistently conceptualized as an extension rather than a replacement of traditional empathy, preserving the relational and humanistic foundations of nursing while adapting empathic practice to technology-mediated care environments. Across the reviewed literature, digital empathy encompassed understanding individuals’ emotional experiences, communicating with compassion, responding to patients’ needs, and maintaining therapeutic relationships through digital technologies while preserving meaningful human connection. Although individual studies emphasized different aspects including authenticity, perspective-taking, emotional regulation, therapeutic presence, cultural sensitivity, digital competence, ethical responsibility, and person-centred care a common conceptual pattern emerged. Collectively, the evidence suggests that digital empathy represents the integration of core empathic nursing attributes with the knowledge, skills, and adaptive capacities required to establish, sustain, and communicate empathic relationships across digitally mediated healthcare settings. Although terminology and theoretical emphasis varied across studies, the synthesized findings revealed substantial conceptual convergence, enabling the development of an integrated conceptual definition of digital empathy presented in Table 4.

**Theme 1: Definition and Conceptualization of Digital Empathy**

Digital empathy was conceptualized in diverse ways across the included studies, with no single definition consistently adopted. Some studies explicitly used terms such as digital empathy, e-empathy, online empathy, virtual empathy, techno-empathy, and augmented caring. Others approached the concept through related constructs, including therapeutic communication, compassionate care, relational presence, trust, authenticity, emotional support, person-centred care, and human-centred communication. Overall, digital empathy was described as the ability to understand, recognize, and respond to individuals’ emotional, psychosocial, and care-related needs within technology-mediated environments while preserving the relational and humanistic values of nursing. Across the literature, it was commonly portrayed as an adaptation of traditional empathy to digital care rather than a completely new phenomenon. Several studies emphasized that empathic understanding can be conveyed despite the absence of physical proximity, particularly through attentive listening, emotional validation, and meaningful responses to individual needs. Digital empathy was also seen as a way of humanizing technology-mediated care by sustaining authenticity, trust, emotional engagement, dignity, and therapeutic relationships. More recently, publications extended the concept to AI-supported care, introducing terms such as techno-empathy, empathic AI, and human–AI collaboration. These studies highlighted that technology should support, rather than replace, empathic communication, professional judgment, and human-centred care.

**Theme 2: Digital Empathy in the Context of Nurse–Individual Interaction and Nursing Care Experience**

The included studies highlighted that digital empathy plays an important role in maintaining nurse–individual relationships and supporting positive care experiences within technology-mediated care environments. Across telehealth, telenursing, telephone consultations, video conferencing, remote monitoring systems, and artificial intelligence-supported care, digital empathy was described as a mechanism for sustaining communication, trust, emotional support, and person-centred care despite physical distance. Several studies emphasized that empathy in digital settings requires nurses to recognize and respond to emotional needs through alternative communication cues when traditional non-verbal signals such as physical presence, touch, and body language are limited. Active listening, timely responses, emotional validation, personalized communication, and therapeutic presence were frequently identified as key components of empathic digital interactions. In addition, digital empathy was associated with increased trust, feelings of safety, reduced loneliness, improved patient satisfaction, and stronger therapeutic relationships. The findings also indicated that technology was generally viewed as a tool for supporting rather than replacing human connection. While telehealth, video consultations, online communication platforms, and artificial intelligence applications may facilitate access to care and continuity of communication, many studies emphasized that empathic nursing care ultimately depends on human understanding, professional judgment, and compassionate engagement.

**Theme 3: Factors Supporting and Hindering Digital Empathy in Technology-Mediated Nursing Care Processes**

The included studies identified individual, educational, technological, organizational, and ethical factors that may facilitate or hinder digital empathy in technology-mediated nursing care. Digital literacy, communication competence, emotional intelligence, active listening, empathic awareness, and the ability to recognize patients’ needs through digital interactions were consistently described as important facilitators. Educational strategies such as simulation, role-play, reflective practice, and digital empathy training were frequently highlighted as approaches for strengthening empathic competencies. User-centred technology design, institutional support, interdisciplinary collaboration, ethical guidance, and nurse involvement in technology development were also identified as important enabling factors. At the same time, the studies reported numerous barriers to digital empathy. The most frequently cited challenges were the absence of physical presence, reduced access to non-verbal communication cues such as body language and touch, technological limitations, internet connectivity problems, and varying levels of digital literacy among patients and healthcare professionals. Concerns related to privacy, confidentiality, data security, algorithmic bias, and the ethical use of artificial intelligence were also widely discussed. Several studies further emphasized the risk of depersonalization, excessive reliance on technology, increased workload, digital fatigue, and the potential weakening of therapeutic relationships when technology becomes the focus rather than a means of supporting person-centred care. The findings suggest that successful implementation of digital empathy depends on balancing technological innovation with the relational, ethical, and human-centred foundations of nursing care.

**Theme 4: Research Gaps and Future Research Needs**

The included studies identified substantial gaps in the current evidence base and highlighted several priorities for future research. A recurring finding was the need for greater conceptual clarity regarding digital empathy and related constructs. Many authors emphasized that digital empathy remains an evolving concept with inconsistent definitions, limited theoretical development, and insufficient understanding of how it differs from or extends traditional empathy within technology-mediated care environments. Another prominent gap concerned education and competency development. Several studies called for the development and evaluation of educational interventions aimed at strengthening digital empathy, communication skills, ethical reasoning, artificial intelligence literacy, and technology-supported therapeutic relationships among nursing students and practicing nurses. The need for structured curricula, simulation-based learning activities, and competency frameworks was frequently emphasized. The literature also highlighted important methodological gaps, particularly the limited availability of valid and reliable instruments specifically designed to measure digital empathy and related constructs in nursing. Many studies recommended the development of culturally sensitive measurement tools, longitudinal research designs, and larger multi-site studies to strengthen the evidence base. Future research was also encouraged to examine the long-term effects of digital empathy on patient outcomes, care experiences, professional wellbeing, and healthcare quality. Finally, numerous studies emphasized the need to explore digital empathy across diverse clinical, cultural, and technological contexts. Particular attention was given to artificial intelligence-supported care, human–AI collaboration, ethical and governance issues, digital inclusion, and the experiences of different patient populations. Across the literature, future research was consistently directed toward ensuring that technological innovation remains aligned with the human-centred and relational foundations of nursing care.

## 4. Discussion

This scoping review synthesized the nursing literature on digital empathy and identified four overarching themes related to its definition and conceptualisation, its role in nurse–individual interactions and nursing care experiences, factors influencing its implementation in technology-mediated care, and research gaps requiring further investigation. Overall, the findings suggest that digital empathy is an evolving concept that has gained increasing attention alongside the expansion of digital health technologies, telehealth services, and artificial intelligence-supported care. Although empathy remains a fundamental component of person-centred nursing practice, the reviewed literature indicates that its expression, communication, and interpretation are being reshaped within digitally mediated care environments. At the same time, persistent conceptual variability, methodological limitations, and research gaps continue to constrain theoretical development and empirical progress in this field. One of the principal findings of this review was the synthesis of a unified conceptualization of digital empathy derived from the included studies. The findings suggest that digital empathy may be understood as a context-dependent extension rather than a replacement of traditional empathy, preserving the relational, ethical, and person-centred foundations of nursing while adapting empathic practice to technology-mediated care through compassionate communication, therapeutic presence, digital competence, and contextual responsiveness. This synthesized conceptualization not only integrates previously fragmented perspectives identified across the literature but also may provide a theoretical foundation to guide future research, educational initiatives, and the implementation of empathic nursing practice in technology-mediated healthcare environments [1,4,12,15,33,35,36]. From a theoretical perspective, the findings further suggest that digital environments may not fundamentally alter the core dimensions of empathy but rather transform how they are expressed in practice. Cognitive empathy may continue to support understanding of patients’ perspectives and needs, affective empathy may remain important for emotional attunement despite reduced physical presence, and behavioural empathy may be expressed through digitally mediated communication, therapeutic responsiveness, and relational presence. These findings suggest that digital technologies primarily reshape the expression of empathic care while preserving its underlying humanistic and relational foundations.

**Theme 1: Definition and Conceptualization of Digital Empathy**

The findings suggest that digital empathy remains an evolving concept with no universally accepted definition in the nursing literature. Included studies used multiple overlapping terms, including digital empathy, e-empathy, techno-empathy, virtual empathy, and augmented caring, while others addressed similar phenomena through related concepts such as therapeutic communication, relational presence, authenticity, emotional support, trust, and person-centred care [1,14,15]. Across studies, digital empathy was generally described as the ability to recognize, understand, and respond to individuals’ emotional and psychosocial needs within technology-mediated encounters while preserving nursing’s relational and humanistic foundations [15,31,36]. Consistent with broader digital empathy literature, most studies conceptualized digital empathy as an adaptation of traditional empathic nursing practices to telehealth, telenursing, online communication, and AI-supported care settings rather than as a fundamentally new phenomenon [27,39,41,46]. A recurring theme across the literature was the view of digital empathy as a human-centred capability that may support trust, compassion, authenticity, and person-centred care within increasingly digital healthcare environments [1,14,15]. However, the conceptual heterogeneity identified across studies creates challenges for theory development, measurement, and comparison of findings. Without clearer conceptual boundaries, it may be difficult to distinguish digital empathy from broader communication competencies or to evaluate interventions designed to strengthen empathic practice in digital settings [14,15]. While concerns remain regarding reduced non-verbal communication, diminished relational closeness, and depersonalized care [19,29,30], the evidence suggests that digital empathy may be understood as a context-dependent extension of traditional empathy that adapts nursing’s humanistic foundations to technology-mediated care environments. Future conceptual development may benefit from greater clarification of how digital empathy differs from related communication and caring constructs while acknowledging variation across care settings and technologies [2].

**Theme 2: Digital Empathy in Nurse–Individual Interaction and the Nursing Care Experience**

Findings across telehealth, telenursing, videoconferencing, and remote monitoring contexts generally highlighted digital empathy as central to sustaining therapeutic nurse–individual relationships. Empathic digital encounters were associated with trust, emotional support, relational presence, reassurance, person-centred communication, and perceived connectedness [17,35,46]. Individuals receiving virtual nursing services reported feeling supported and connected when nurses demonstrated attentiveness, responsiveness, and relational engagement during digital interactions [49,50]. Collectively, these findings suggest that empathy remains a fundamental component of nursing care regardless of the communication medium and may be conveyed through digital platforms. At the same time, the studies suggest that digital care environments transform rather than merely replicate traditional nurse–individual interactions. Technology may reduce access to certain non-verbal cues, limit opportunities for embodied presence, and introduce interactional challenges that influence how empathy is perceived and communicated [41,42,46]. Such changes may require nurses to adapt their communication strategies to convey presence, understanding, and emotional support. Importantly, these findings suggest that digital empathy may be understood not only as an individual nurse attribute but also as a relational and context-dependent phenomenon shaped by technology design, workflow constraints, and the broader care environment. Nevertheless, the reviewed literature suggests that the quality of digital nurse–individual interactions appears to depend less on the technology itself and more on how effectively digital tools are used to support human connection, therapeutic relationships, and person-centred care [31,32]. The findings also suggest that the communication modality itself may influence opportunities for empathic engagement. When feasible, video-based interactions may provide greater access to visual and relational cues than audio-only communication, potentially supporting empathic understanding and relational presence [39,50]. However, when video communication is not possible, the effective use of verbal communication, including tone of voice, pacing, active listening, and emotional validation, may become particularly important for conveying empathy in digital encounters [35]. These considerations reinforce the importance of preparing nurses to adapt empathic communication strategies to different digital modalities while maintaining the person-centred and relational foundations of nursing care.

**Theme 3: Factors Supporting Digital Empathy in Technology-Mediated Nursing Care**

The findings of this review suggest that the development and maintenance of digital empathy appear to depend on interacting individual, technological, and organizational factors. At the individual level, digital competence, communication skills, adaptability, and confidence in using digital technologies were consistently identified as important facilitators of establishing empathic relationships in technology-mediated care environments [14,15,16]. Several studies emphasized that nurses may require not only technical proficiency but also the ability to adapt traditional empathic practices to digital channels by conveying attentiveness, emotional presence, and compassion through verbal communication and other available relational cues [35,36]. Beyond digital competence and communication skills, individual psychological capabilities may also influence the expression of digital empathy. Although these factors were not extensively examined in the included studies, broader nursing literature suggests that effective emotion regulation may play an important role in adaptive coping and sustaining empathic functioning in demanding healthcare environments [51,52]. Furthermore, nurses’ emotional well-being and regulation have been associated with resilience, professional adaptation, quality of interpersonal care, and patient safety [53,54]. In addition, evidence suggests that psychological flexibility has been associated with emotional intelligence and emotion regulation, highlighting the importance of considering these psychological processes alongside technological and organizational factors when developing digital empathy in technology-mediated healthcare settings [51,55]. Emerging evidence also suggests that self-compassion and positive mental health may strengthen empathic engagement by supporting nurses’ emotional resources and psychological well-being, thereby promoting more sustainable therapeutic relationships [7]. Furthermore, compassionate care in digital environments may depend not only on technological competence but also on communication strategies that are responsive to patients’ needs, supported by adequate training, institutional support, and consideration of patients’ eHealth literacy [35]. At the technological and organizational levels, human-centred design, accessible platforms, interprofessional collaboration, leadership support, digital literacy initiatives, and opportunities for training were identified as important facilitators of empathic care [1,19,32,56]. Several studies also suggested that the way digital empathy is understood and enacted may vary across cultural settings, care contexts, and healthcare systems, highlighting the importance of context-sensitive approaches rather than assuming a single universal model of empathic digital care [2,31]. Conversely, several barriers were identified that may hinder the development and expression of digital empathy. Technical interruptions, usability challenges, limited non-verbal communication, insufficient training, workload pressures, and poorly integrated digital systems were frequently reported as factors that may weaken therapeutic relationships and reduce opportunities for meaningful interpersonal engagement [28,42]. In addition, concerns related to depersonalization, excessive reliance on technology, digital fatigue, and inequitable access to digital health services were discussed across the literature [18,19]. Ethical considerations, including privacy and data security, algorithmic bias, and the responsible use of artificial intelligence, were also identified as important issues that may influence trust, authenticity, and relational safety within technology-mediated care environments [12,18]. Taken together, these findings suggest that digital empathy is not supported by technology alone but appears to emerge through the interaction of digital competence, empathic communication, human-centred design, organizational support, and ethical implementation of digital health technologies. Across the reviewed studies, technology was generally viewed as a means of supporting rather than replacing human connection, reinforcing the importance of preserving the relational and humanistic foundations of nursing within increasingly digital healthcare environments [2,18,51].

**Theme 4: Research Gaps and Future Research Needs in the Digital Empathy Literature**

The included studies identified substantial gaps in the current evidence base. A recurring finding was the need for greater conceptual clarity regarding digital empathy and related constructs. Many authors emphasized that digital empathy remains an evolving concept with inconsistent definitions, limited theoretical development, and insufficient understanding of how it differs from or extends traditional empathy within technology-mediated care environments [14,15]. This conceptual heterogeneity continues to present challenges for theory development, comparison of findings, and the advancement of a coherent body of knowledge in the field. Another prominent gap concerned education, competency development, and measurement. Several studies called for the design and evaluation of educational interventions aimed at strengthening digital empathy, communication skills, ethical reasoning, artificial intelligence literacy, and digital professionalism [16,33]. At the same time, the literature continues to lack robust, context-sensitive instruments capable of assessing digital empathy across different technologies, care settings, and populations [14,15]. Recent conceptual work has also highlighted the need to move beyond technology-focused definitions toward theoretically grounded frameworks that integrate humanistic nursing values with technological competency. In this context, the emerging concept of techno-caritas has been proposed as a broader conceptual framework that positions digital empathy within the ethical, relational, and caring dimensions of technology-mediated nursing practice, emphasizing that technology should extend rather than replace therapeutic relationships [12,56]. Such conceptual advances may provide an important foundation for the future development of valid and theoretically informed measurement instruments while helping to clarify the boundaries between related constructs such as digital empathy, digital compassion, and techno-caritas. Without reliable measurement approaches, it may remain difficult to evaluate the effectiveness of educational interventions or to examine the relationship between digital empathy and outcomes such as trust, satisfaction, therapeutic relationships, quality of care, and professional well-being [17,50]. In addition to developing novel instruments, future research should also examine whether existing validated empathy measures used in healthcare and health professions education can be adapted to digitally mediated care contexts. Such adaptation would require careful evaluation of their content validity, construct validity, and psychometric performance to determine whether they adequately capture the unique relational, communicative, and technological dimensions of digital empathy. The reviewed studies also highlighted the need to expand research across diverse clinical, cultural, and technological contexts. Several authors emphasized the importance of examining digital empathy within AI-supported care, human–AI collaboration, governance and accountability processes, and efforts to promote digital inclusion [2,18,31]. Future research should also examine how cultural values, communication norms, healthcare systems, and levels of digital maturity influence the expression, perception, and implementation of digital empathy across diverse populations and care settings. Such evidence may contribute to the development of culturally sensitive conceptual frameworks, educational strategies, and measurement instruments for digital empathy. Furthermore, concerns related to equity, digital literacy, access to technology, and the ethical use of artificial intelligence suggest that future research should consider how digital empathy is experienced and sustained among different populations and healthcare systems [4,33,36].

Overall, the findings of this review suggest that digital empathy represents an evolving extension of traditional empathic nursing practice rather than a replacement for it. While digital technologies may create new opportunities to maintain therapeutic relationships, improve access to care, and support patient engagement, they also introduce challenges related to communication, relational presence, professional identity, equity, and the ethical use of technology. Future research may benefit from strengthening conceptual foundations, developing robust measurement approaches, evaluating educational interventions, and generating context-sensitive evidence across diverse populations, care settings, and digital health systems. Such efforts may contribute to a more comprehensive understanding of how digital empathy can be effectively taught, implemented, sustained, and evaluated within contemporary nursing practice.

### 4.1. Strengths

This scoping review has several notable strengths. The review protocol was prospectively registered on the Open Science Framework, enhancing transparency and methodological rigor. The review was conducted in accordance with the Joanna Briggs Institute methodology for scoping reviews and reported following the PRISMA-ScR guideline. A comprehensive search strategy was implemented across four major databases, and study selection and data extraction were conducted using independent dual-reviewer procedures with oversight from a third reviewer, helping to enhance consistency and minimize selection bias. In addition, this review synthesizes evidence from diverse clinical, educational, cultural, and technological contexts, providing a broad overview of how digital empathy has been conceptualized and discussed within contemporary nursing literature. By mapping the breadth and characteristics of the available evidence, the review may contribute to a clearer understanding of the current state of knowledge and the remaining gaps in this emerging field.

### 4.2. Limitations

Several limitations of this scoping review should be acknowledged. First, only studies published in English or Turkish were included, which may have resulted in the exclusion of relevant evidence published in other languages. Although these languages were selected because they were the only languages in which the review team could reliably screen, extract, and interpret data without translation, potentially relevant studies from other linguistic and regional contexts may not have been captured, thereby limiting the comprehensiveness and international representativeness of the evidence base. Second, although a comprehensive search strategy was implemented across four major databases, relevant studies indexed exclusively in databases from related disciplines (e.g., psychology, communication, or behavioral sciences), as well as unpublished evidence, may not have been captured. Consequently, some interdisciplinary perspectives on digital empathy may be underrepresented in the mapped evidence base. In addition, grey literature, conference abstracts, theses, book chapters, and other non-peer-reviewed sources were excluded, which may have limited the identification of emerging perspectives in this rapidly evolving field. Furthermore, the evolving and terminologically heterogeneous nature of digital empathy means that some relevant studies may not have been identified despite efforts to incorporate a broad range of related search terms. Publication bias may also have influenced the available evidence, as studies reporting positive findings may be more likely to be published than those reporting null or negative results. In addition, most included studies were conducted in high-income countries, which may limit the transferability of the findings to settings with different healthcare systems, digital infrastructures, and cultural contexts. Finally, consistent with the purpose and methodology of a scoping review, a formal methodological quality appraisal of the included studies was not conducted. Therefore, the findings should be interpreted as a mapping of the available evidence rather than an evaluation of the quality, validity, or strength of that evidence. The findings of this review reflect the state of the literature available up to April 2026 and should be interpreted within this context.

### 4.3. Implications

The findings of this review suggest that digital empathy is becoming an increasingly relevant component of contemporary nursing practice as healthcare delivery continues to expand across technology-mediated environments. The reviewed literature indicates that empathic care in digital settings involves not only traditional interpersonal competencies but also the ability to adapt communication practices to evolving digital technologies. Consequently, digital empathy may represent an important consideration for nursing education and professional development. The findings further highlight the potential value of educational approaches that support communication skills, reflective practice, digital professionalism, ethical reasoning, and artificial intelligence literacy in preparing nurses for technology-mediated care environments [16,33,51]. The review also indicates that digital empathy is influenced by factors extending beyond individual nurses’ communication skills. Human-centred technologies, organizational support, accessibility, digital literacy, and ethical considerations were frequently discussed as important elements shaping empathic interactions in digital care settings [1,19,51]. In addition, the reviewed studies emphasized the importance of designing digital health technologies that support meaningful relational engagement while ensuring equitable access and responsible use of emerging technologies, including artificial intelligence [2,50]. The reviewed literature further suggests that the communication modality itself may influence opportunities for empathic engagement. When feasible, video-based interactions may provide additional visual and relational cues that support empathic communication, whereas audio-only encounters may require greater reliance on verbal elements such as tone of voice, pacing, active listening, and emotional validation [35,39,50]. These findings highlight the importance of preparing nurses to adapt empathic communication strategies across different digital care modalities and suggest that structured approaches to communication may be valuable in supporting relational and person-centred care in technology-mediated nursing environments. Collectively, these findings suggest that the successful integration of digital empathy into nursing practice may depend not only on individual competencies but also on the broader technological, organizational, and ethical contexts in which care is delivered.

## 5. Conclusions

This scoping review mapped the literature on digital empathy in nursing and identified how the concept has been defined, experienced, supported, and challenged within technology-mediated care environments. The findings suggest that digital empathy is increasingly recognized as an important component of person-centred digital care and may be understood as an evolving extension of traditional empathic nursing practice. Across the reviewed studies, digital empathy was characterized as a multidimensional and context-dependent concept shaped by individual competencies, technological characteristics, organizational conditions, and broader ethical and cultural influences. However, conceptual definitions remain inconsistent, theoretical development remains limited, and validated measurement approaches are still lacking. The review also identified important gaps in the current evidence base. Future research may benefit from strengthening the conceptual foundations of digital empathy, developing robust and context-sensitive measurement instruments, and evaluating educational, organizational, technological, and communication-based approaches that support empathic care in digital settings. Particular attention may also be given to how empathic communication can be effectively adapted across different digital modalities, including video- and audio-based care encounters, while preserving the relational and person-centred foundations of nursing care. Particular attention may also be given to understanding how digital empathy influences therapeutic relationships, patient outcomes, professional well-being, and quality of care across diverse clinical, cultural, and technological contexts. Overall, the findings suggest that digital empathy may represent an important area of development for contemporary nursing as healthcare systems continue to integrate digital technologies and artificial intelligence into care delivery. Continued research and innovation may contribute to ensuring that technological advancement is accompanied by the preservation of the relational, compassionate, and human-centred foundations of nursing practice.

## Figures and Tables

**Table 1:** Search strategies used in the electronic databases.

Database	Research Strategy
PubMed	(nurs*[Title/Abstract] OR “nursing student*”[Title/Abstract] OR “nursing care”[Title/Abstract] OR “nurse-patient relation*”[Title/Abstract]) AND (empath*[Title/Abstract] OR “empathic communication”[Title/Abstract] OR “empathic care”[Title/Abstract]) AND (telehealth[Title/Abstract] OR telemedicine[Title/Abstract] OR digital[Title/Abstract] OR online[Title/Abstract] OR virtual[Title/Abstract] OR “remote care”[Title/Abstract] OR “digital communication”[Title/Abstract] OR “online communication”[Title/Abstract] OR “computer-mediated communication”[Title/Abstract])
Scopus	(nurs* OR “nursing student*” OR “nursing care” OR “nurse-patient relation*”) AND (empath* OR “empathic communication” OR “empathic care”) AND (telehealth OR telemedicine OR digital OR online OR virtual OR “remote care” OR “digital communication” OR “online communication” OR “computer-mediated communication”)
CINAHL	(TI nurs* OR AB nurs* OR TI “nursing student*” OR AB “nursing student*” OR TI “nursing care” OR AB “nursing care” OR TI “nurse-patient relation*” OR AB “nurse-patient relation*”) AND (TI empath* OR AB empath* OR TI “empathic communication” OR AB “empathic communication” OR TI “empathic care” OR AB “empathic care”) AND (TI telehealth OR AB telehealth OR TI telemedicine OR AB telemedicine OR TI digital OR AB digital OR TI online OR AB online OR TI virtual OR AB virtual OR TI “remote care” OR AB “remote care” OR TI “digital communication” OR AB “digital communication” OR TI “online communication” OR AB “online communication” OR TI “computer-mediated communication” OR AB “computer-mediated communication”)
Web of Science	(nurs* OR “nurse-patient”) AND (empath* OR “digital empathy” OR “e-empathy”) AND (tele* OR digital OR online OR virtual OR eHealth OR mHealth OR “mobile health” OR “remote care” OR “remote monitoring” OR videoconferenc* OR “computer-mediated”)

**Table 2:** Characteristics of Included Studies.

No	Author (Year)	Country	Study Type	Population/Context	Digital Setting/Technology	Main Focus
**1**	Rabe (2025) [14]	USA	Concept analysis	Nursing education	Digital communication, telehealth, AI, virtual environments	Conceptualization and attributes of digital empathy in nursing education
**2**	Gutiérrez-Puertas et al. (2024) [16]	Spain	Comparative cross-sectional study	Nursing students	Telephone consultation, videoconferencing	Communication and empathy in telenursing education
**3**	Šimunić et al. (2026) [26]	Croatia	Narrative review	Oncology nursing education and practice	Artificial intelligence, chatbots, virtual assistants, decision-support systems	AI literacy, digital competence, and human-centered oncology nursing care
**4**	Ali & Shaban (2026) [2]	Saudi Arabia	Qualitative study	Older adults and nurses in virtual communities	Videoconferencing, online communication platforms	Nurses’ role in fostering social connectedness and emotional support in virtual communities
**5**	Skogevall et al. (2021) [27]	Sweden	Cross-sectional survey	Nurses	Telephone nursing services	Relationships among empathy, stress, and self-efficacy in telephone nursing
**6**	Torres-Vigil et al. (2021) [17]	USA	Qualitative study	Advanced cancer patients receiving palliative care	Telephone-based nursing intervention	Empathic communication and therapeutic support in telephone nursing
**7**	Staats et al. (2026) [4]	Norway/Singapore	Scoping review	Home-dwelling older adults and nurses	Telemonitoring, videoconferencing, mobile applications, digital communication platforms	Digital communication services supporting nurse–patient dialogue in home care
**8**	Günday & Güler (2026) [20]	Türkiye	Qualitative phenomenological study	Psychiatric nurses	Artificial intelligence applications in mental health care	Nurses’ perceptions, concerns, and readiness regarding AI in psychiatric nursing
**9**	Pepito et al. (2026) [15]	Philippines/Saudi Arabia/Canada	Conceptual paper	Technology-mediated nursing care environments	Telehealth, EHRs, AI-supported systems, remote monitoring	Development of a situated model of digital empathy in nursing
**10**	Abou Hashish & Alnajjar (2025) [28]	Saudi Arabia	Concept analysis	Digital nursing and telehealth contexts	Telehealth, virtual care platforms, AI-supported triage systems	Conceptualization of digital compassion fatigue in nursing
**11**	Knop et al. (2024) [19]	Germany	Literature review	Clinical nursing settings	EHRs, telehealth, monitoring systems, digital health technologies	Impact of digital technologies on nursing professional identity, power relations, and empathic care
**12**	Wynn (2024) [29]	UK	Conceptual/critical discussion	Nurses, nursing students, care recipients	Digital health technologies, care robots, AI-supported care	Re-conceptualizing caring, empathy, and human-centered nursing in the digital age
**13**	Luengo Polo et al. (2025) [30]	Spain	Qualitative study	Primary care nurses	ACHO virtual voice assistant	Nurses’ perceptions of digital assistants for treatment adherence and their impact on nurse–patient relationships
**14**	Nashwan & Kunjavara (2026) [31]	Qatar	Theoretical/conceptual paper	AI-supported nursing care	AI, machine learning, decision support systems, telehealth	Preserving empathy and the human essence of nursing through techno-empathy and augmented caring
**15**	Girdwood et al. (2026) [32]	USA	Perspective/conceptual paper	Cancer care navigation	Empathic AI, agentic AI, chatbots, virtual assistants	Human-centered framework for integrating empathic AI into nursing and cancer care navigation
**16**	Badawy et al. (2025) [33]	Saudi Arabia	Qualitative study	Registered nurses in AI-supported clinical settings	AI decision-support systems, predictive analytics	Ethical boundaries, data sharing, empathy, and human interaction in AI-enhanced nursing
**17**	Pepito et al. (2026) [18]	Philippines/Australia/Bangladesh authors	Discursive/conceptual paper	Nursing education	AI, telehealth, AI-enhanced simulation, remote monitoring	Human–AI collaboration competencies and digital empathy in nursing curricula
**18**	Monteiro et al. (2017) [34]	Portugal	Theoretical paper	Technology-intensive nursing care	Biotechnology, digital health technologies	Preserving aesthetic knowledge, therapeutic presence, and humanized care in digital environments
**19**	Su et al. (2024) [35]	China- Hong Kong	Qualitative study (IPA)	Nurses and physicians providing eHealth services	Telehealth, video consultations, mobile apps, social media platforms	Experiences of delivering compassionate care and empathy through eHealth
**20**	Abou Hashish (2025) [1]	International conceptual perspective	Concept analysis	Telehealth nursing and digital care	Telehealth, telemonitoring, messaging platforms, AI-supported sentiment tools	Defining digital empathy, identifying attributes, antecedents, barriers, and outcomes
**21**	Glenn et al. (2025) [36]	USA	Qualitative case study	Black patients with chronic kidney disease	Telehealth, Zoom, e-empathy guide	Development and adaptation of culturally sensitive e-empathy in goals-of-care conversations
**22**	Grinberg & Sela (2023) [37]	Israel	Cross-sectional study	Nurses	Telenursing (phone, email, chat, video)	Comparison of perceived quality of telenursing and face-to-face nursing care
**23**	Johnson et al. (2026) [38]	UK/USA	Discussion paper	ICU survivors and families	Generative AI, digital recovery narratives	Humanizing post-ICU recovery through AI-supported empathetic storytelling
**24**	Koppel et al. (2022) [39]	USA	Qualitative descriptive study	Oncology patients and nurses	Video consultations, telehealth	Building rapport and person-centred relationships during oncology video visits
**25**	Li et al. (2025) [40]	International	Scoping review	Cancer patients	AI conversational agents, chatbots	Usability, patient experience, emotional support, and empathy limitations of chatbots in cancer education
**26**	Solli & Hvalvik (2019) [41]	Norway	Qualitative study	Nurses supporting caregivers of people with stroke/dementia	Telecare, webcam, web forum	Providing empathy, support, and relational care at a distance
**27**	Nagel et al. (2013) [42]	Canada	Discussion paper	Telehealth nursing practice	Telehealth, videoconferencing, remote monitoring	Caring, knowing, and maintaining holistic nursing care through telehealth
**28**	Snelgrove (2009) [43]	UK	Qualitative study	NHS Direct nurses	Telephone-based nursing, call-centre systems	Construction of nursing identity and holistic care in remote communication
**29**	Vaz et al. (2023) [44]	India	Qualitative study	ICU nurses and intensivists during COVID-19	Mobile phones, video calls, messaging applications	Challenges of maintaining empathetic communication during crisis care
**30**	Enam et al. (2022) [45]	Norway	Qualitative case study	Nurses, patients, GPs, managers	Distance monitoring, telecare platforms	Co-creation of care and nurses’ role as empathetic listeners in remote monitoring
**31**	Grimsbø et al. (2012) [46]	Norway	Quantitative content analysis	Cancer patients and oncology nurses	Online patient–nurse communication, e-mail messaging	Emotional cues and empathic responses in online nurse–patient communication
**32**	Bjorklund (2016) [47]	USA	Commentary paper	Nursing, education, society	Digital technologies, social media, internet	Impact of digital technology on empathy, attention, and relational care
**33**	Metcalfe & Putnam (2013) [48]	USA	Discussion paper	Nursing students and educators	Electronic communication, online learning	Preserving empathetic communication in the digital era

**Table 3:** Methodological Characteristics of the Included Studies.

Methodological Design	Number of Studies (n)	Percentage (%)
**Qualitative studies**	12	36.4
**Quantitative studies**	4	12.1
**Concept analyses**	3	9.1
**Reviews**	4	12.1
**Theoretical/conceptual/discussion papers**	10	30.3
**Total**	33	100.0

**Table 4:** Synthesized Conceptual Definition and Core Components of Digital Empathy Identified Across the Included Studies.

Conceptual Component	Synthesized Description	Supporting Evidence
**Conceptual definition**	Digital empathy is the integration of empathic nursing attributes with technology-mediated communication, enabling nurses to understand, communicate, and respond compassionately to individuals’ emotional and relational needs while preserving meaningful human connection across digital healthcare environments.	Consistently synthesized from concept analyses and empirical studies examining digital, virtual, telehealth, AI-supported, and remote nursing care.
**Emotional understanding**	Recognizing patients’ emotions, concerns, values, and lived experiences despite reduced or altered interpersonal cues in digital environments.	Frequently identified across telehealth, telenursing, videoconferencing, and qualitative studies exploring nurse-patient communication.
**Compassionate communication**	Conveying empathy through active listening, emotional validation, supportive verbal or written communication, and compassionate responses using digital communication technologies.	Consistently reported across studies examining remote communication, telehealth consultations, eHealth services, and digital nursing interactions.
**Therapeutic relationship and human connection**	Establishing trust, authenticity, therapeutic presence, rapport, and relational continuity while maintaining meaningful human connection in technology-mediated care.	Commonly emphasized across studies exploring virtual nurse–patient relationships, technology-mediated caring, and digital communication.
**Person-centred care**	Respecting individual preferences, dignity, autonomy, cultural context, and shared decision-making while adapting care to patients’ needs in digital environments.	Frequently discussed in studies addressing telehealth, community care, older adult care, culturally sensitive care, and compassionate digital practice.
**Digital competence and contextual adaptation**	Integrating empathic communication with digital literacy, technological competence, adaptability, and context-sensitive use of digital health technologies.	Predominantly highlighted in educational studies, concept analyses, AI-related publications, and digital health competency literature.
**Ethical and professional responsibility**	Preserving privacy, confidentiality, transparency, equity, professional accountability, and responsible use of emerging technologies, including artificial intelligence, while maintaining the humanistic values of nursing.	Consistently emphasized in publications focusing on AI-supported nursing, digital ethics, governance, and responsible technology implementation.
**Overall conceptualization**	The synthesis suggests that digital empathy represents a context-dependent extension rather than a replacement of traditional empathy, preserving the relational, ethical, and person-centred foundations of nursing while adapting empathic practice to technology-mediated healthcare environments.	Synthesized across all included conceptual, empirical, educational, and discussion papers addressing empathy within digitally mediated nursing practice.

## Data Availability

No new data were created or analyzed in this study. Data sharing is not applicable to this article.
